# Fungal infections: Pathogenesis, antifungals and alternate treatment approaches

**DOI:** 10.1016/j.crmicr.2022.100137

**Published:** 2022-04-27

**Authors:** G.Kiran Kumar Reddy, Alwar Ramanujam Padmavathi, Y.V. Nancharaiah

**Affiliations:** a Biofouling and Biofilm Processes Section, Water and Steam Chemistry Division, Chemistry Group, Bhabha Atomic Research Centre, Kalpakkam 603 102, Tamil Nadu, India; b Homi Bhabha National Institute, Anushakti Nagar, Mumbai 400 094, India

**Keywords:** Antifungal resistance, Alternate antifungal therapies, Biofilm, Fungal pathogenesis, Host-immune response, ABC, ATP-Binding Cassette, APC, Antigen Presenting Cells, BBB, Blood Brain Barrier, BAD1, Blastomyces Adhesin-1, CDC, Centers for Disease Control and Prevention, CNS, Central Nervous System, DC, Dendritic Cells, ERG, ETS-Related Gene, ETS, Erythoblast Transformation Specific, FCY, Fluorocytosine Deaminase, FDA, Food and Drug Administration, G-CSF, Granulocyte-Colony Stimulating Factor, GM-CSF, Granulocyte-Macrophage Colony Stimulating Factor, HAI, Healthcare Associated Infections, HIV, Human Immunodeficiency Virus, Hsp, Heat Shock Protein, IFN, Interferon, MFS, Major Facilitator Superfamily, M-CSF, Macrophage-Colony Stimulating Factor, NK, Natural Killer, PRR, Pattern Recognition Receptors, PAMP, Pathogen Associated Molecular Pattern, ROS, Reactive Oxygen Species, RNS, Reactive Nitrogen Species, TLR, Toll-Like Receptors

## Abstract

Increasing incidence of fungal infections of recent times requires immediate intervention. Fungal infections are seldom construed at initial stages that intensify the severity of infections and complicate the treatment procedures. Fungal pathogens employ various mechanisms to evade the host immune system and to progress the severity of infections. For the treatment of diverse superficial and systemic infections, antifungal drugs from the available repertoire are administered. However, well documented evidence of fungal resistance to most of the antifungal drugs hampers disease control and poses challenges in antifungal therapy. Several physiological adaptations and genetic mutations followed by their selection in presence of antifungal agents drive the resistance development in fungi. The availability of limited antifungal arsenal, emergence of resistance and biofilm-conferred resistance drives the need for development of novel drugs and alternate approaches for the better treatment outcome against mycoses. This graphical review explicitly shed light on various fungal infections and causative organisms, pathogenesis, different antifungal drugs and resistance mechanisms including host immune response and evasion strategies. Here, we have highlighted recent developments on novel antifungal agents and other alternate approaches for fighting against fungal infections.

## Graphical abstract

**Figure:**
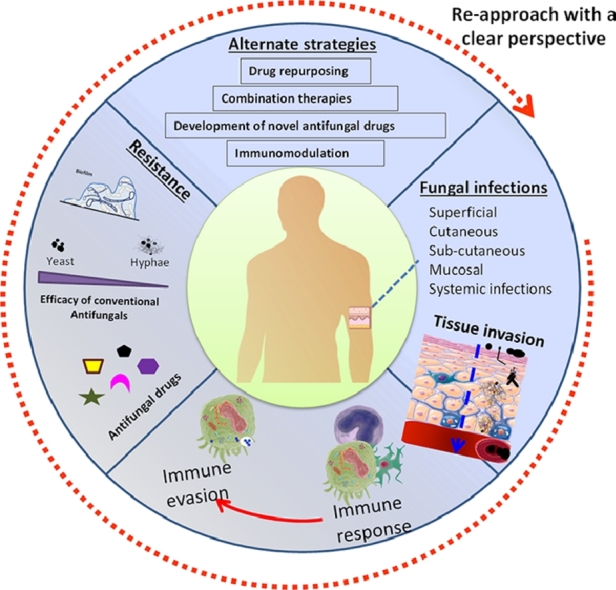

## 1 Introduction

Fungal infections are of serious public health concern. The incidence of fungal infections in patients with other diseases including Covid-19 is associated with life-threatening mycoses and mortality. Fungal infections can include superficial, cutaneous, sub-cutaneous, mucosal and systemic infections with varying degree of severity. Organisms such as *Candida* spp. are part of human microbiota that can cause opportunistic infections in individuals and life threatening infections (invasive candidiasis) in immuno-compromised patients such as HIV patients, cancer patients receiving chemotherapy, and patients receiving immuno-suppressive drugs. Besides, opportunistic and systemic infections, fungal pathogens such as *Candida, Aspergillus, Fusarium,* Mucorales and molds can cause healthcare-associated infections (HAI) in patients with underlying diseases (Perlroth et al., 2007). In certain geographical areas, fungal pathogens cause prevalent life-threatening endemic mycoses such as Blastomycosis, Coccidiodomycosis, Histoplasmosis, Talaromycosis, Paracoccidiodomycosis and Sporotrichosis (Lee and Lau, 2017).

Systemic fungal infections are often diagnosed lately increasing mortality rates. Centers for Disease Control and Prevention (CDC) has declared September 20–24, 2021 as fungal disease awareness week, to educate and to highlight the importance of early diagnosis of fungal infections to alleviate the debilitating effects (CDC website). This article provides an overview of the spectrum of fungal infections in humans, pathogenesis, immune evasion mechanisms, antifungal drugs along with their mode of action, resistance mechanisms and alternate antifungal approaches to combat fungal infections.

## 2 Fungal pathogens and routes of transmission

**Fig. 1:**
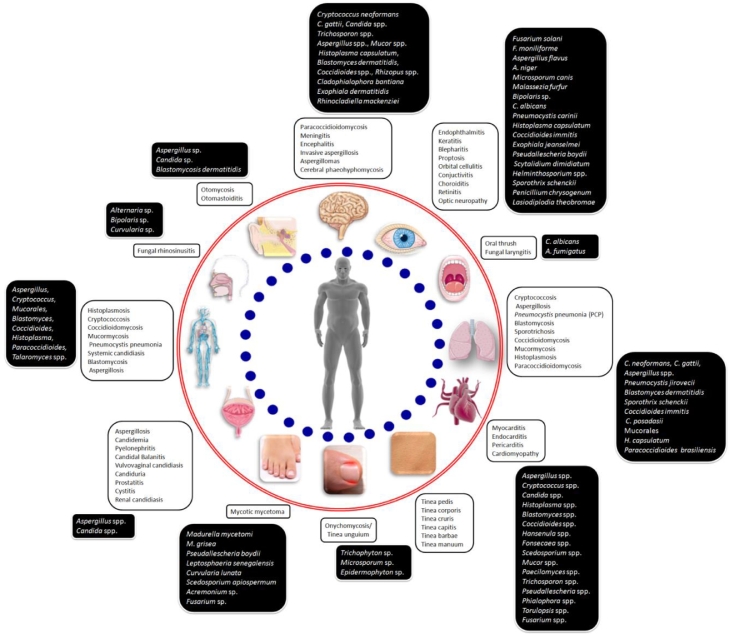
Spectrum of fungal infections and their etiological agents in humans.

The sub-kingdom Dikarya of fungi comprising of the phyla Ascomycota and Basidiomycota is the major contributor of all fungal pathogens and infections in humans. Ascomycota organisms are known for causing oropharyngeal, otolaryngeal, dermatological, ophthalmic, neuronal, genitourinary, cardiac, pulmonary and systemic infections (Fig. 1). The organisms of Basidiomycota such as *Cryptococcus* and *Malassezia* are well-known for invasive meningitis and superficial skin infections, respectively.

**Fig. 2:**
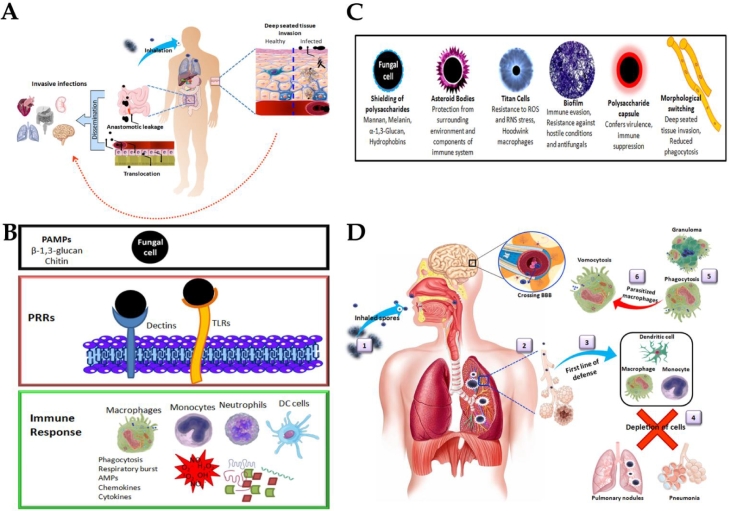
**A.** Routes of invasion of fungal pathogens. **B.** Pathogen recognition and response in host immune system. **C.** Morphological modulation of fungal cells for immune evasion. **D.** Pulmonary transmission and pathogenesis of invasive fungal infections. 1. Inhalation of spores or conidia; 2. Entry into alveoli; 3. Eliciting first line of defense; 4. Depletion of phagocytic cells leads to disease progression as pulmonary nodules and pneumonia; 5. Macrophages phagocytise the fungal cells or encapsulate and form granuloma; 6. Fungal cells parasitize the macrophages that leads to vomocytosis of intact fungi and circulation into bloodstream and crossing blood brain barrier to cause systemic infections.

Fungal pathogens primarily use direct contact and/or inhalation route for transmission. Dermatophytic fungi belonging to the genera of *Microsporum, Epidermophyton* and *Trichophyton, Sporothrix* and *Malassezia* spp. infect the damaged skin by direct contact (Fig. 2A).They produce various proteolytic enzymes to cause superficial mycoses in keratinized tissues. The other predominant route for transmission is by inhalation of spores/ conidia that instigates pulmonary infections. *Blastomyces dermatitidis* (Blastomycosis), *Paracoccidioides brasiliensis* and *P. lutzii* (Paracoccidiodomycosis), *Histoplasma capsulatum* (Histoplasmosis), *Pneumocystis jirovecii* (Pneumocystis pnuemonia), *Aspergillus fumigatus* and *A. flavus* (Aspergillosis), *Coccidioides immitis* and *C. posadasii* (Coccidioidomycosis), *C. neoformans* and *C. gattii* (Cryptococcosis) are mainly transmitted through inhalation. While, *Talaromyces marneffei* (talaromycosis) uses both direct contact and inhalation route for transmission.

## 3 Arsenals of host immune system and fungal immune evasion

Host immune system contains pattern recognition receptors (PRR) like dectins and Toll-like receptors (TLR) that recognize pathogen-associated molecular pattern (PAMP) components of fungal cell wall such as chitin and *β*−1,3-glucans. This recognition of PAMP by PRRs triggers signal transduction pathways that lead to generation of an immune response involving phagocytosis, respiratory burst of reactive oxygen species (ROS), reactive nitrogen species (RNS), production of cytokines and chemokines that elicit strong inflammatory response for neutralizing fungal pathogen (Fig. 2B). Monocytes, macrophages, dendritic cells (DC) and neutrophils act as the first line of defense in innate immune response. The fungal cells can be eliminated at phagolysosome and contained as granulomas surrounded by macrophages. Monocytes secrete chemokines and cytokines in addition to acting as antigen presenting cells (APC) to T-lymphocytes to elicit adaptive immune response that helps in clearing fungal cells. Fungal pathogens follow various survival mechanisms to thrive and hoodwink host immune system (Fig. 2C). Fungal cells modulate their surface layers to shield the highly conserved PAMPs to prevent recognition by PRRs (Campuzano et al., 2018). *P. jirovecii* has major surface glycoprotein that masks surface *β*−1,3-glucan and the cell wall is lack of chitin (Ma et al., 2016). Fungal mannan, melanin, *α*−1,3-glucan, and hydrophobin (*Aspergillus* RodA) layers shield the surface polysaccharides that act as PAMPs, thereby evade the host immune system (Hernández-Chávez et al., 2017). The surface *β*−1,3-glucan segments exposed on *H. capsulatum* surface were trimmed by endo *β*−1,3-glucanase causing a reduction in pathogen recognition and stimulation of proinflammatory cytokines (Garfoot et al., 2016). Besides surface shielding, capsular polysaccharide of *C. neoformans* confers virulence and adversely affects T cell activation and neutrophil recruitment. Fungal pathogens form morphologically different structures for evading immune recognition. For example, *C. neoformans* forms titan cells (Zaragoza, 2011) and *S. schenckii* forms asteroid bodies (Rosa et al., 2008). Some fungal pathogens adapt dimorphic growth for virulence and immune evasion. For example, *Candida* spp. grows in yeast form and switches to form filamentous hyphae that help in disease onset and progression. This morphological switching has the advantages of increased tissue invasion and reduced phagocytosis besides bursting the membrane of the phagocytic cell (Marcos et al., 2016). *C. immitis* and *C. posadasii* can undergo morphological switching from arthroconidia to spherule state which is associated with an outer cell wall glycoprotein and production of enzymes such as arginase and urease (Diep and Hoyer, 2020). Spherules are the reproductive form of fungus in the host and exhibit resistance to phagocytosis and RNS.

Some fungi can parasitize the macrophages and escape from phagocytosis (Zhi et al., 2019). For example, *C. neoformans* infect the lungs and depletes the alveolar macrophages (Fig. 2D). It parasitizes the macrophages that leads to vomocytosis which release intact fungal cells to cross the blood-brain barrier (BBB) and affect brain, cerebrospinal fluid, central nervous system (CNS) and manifests cryptococcal meningitis (Elsegeiny et al., 2018). *B. dermatitidis* spores can also evade the immune system in a similar way and circulate in lymphatic system and blood. Blastomyces adhesin-1 (BAD1) protein inactivates complement cascade and escalates systemic infections. Overall immune evasion gives the benefit of anti-phagocytic effect, suppression of T cell proliferation, proinflammatory cytokines and resistance to oxidative and nitrosative stresses.

In addition to immune evasion, biofilm formation in fungal pathogens (e.g., *C. albicans, C. neoformans, H. casulatum, P. brasiliensis* and *A. fumigatus*) provides seminal advantage of thriving under hostile environments and evading antifungal treatments.

## 4 Antifungal agents and their mechanism of action

**Fig. 3:**
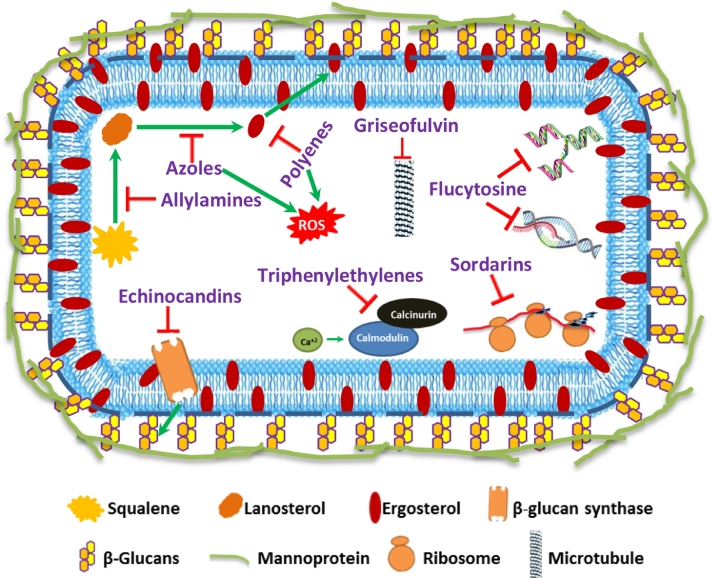
Existing and new cellular targets including mode of action of antifungal drugs.

Currently, five common classes of antifungal drugs such as azoles, polyenes, echinocandins, allylamines and pyrimidine analogs are available for superficial and systemic antifungal therapies (Hokken et al., 2019). The mechanism of action along with potential cellular targets for these drugs is depicted in Fig. 3. Among these, azole class of drugs includes imidazoles (miconazole and ketoconazole) and triazoles (fluconazole and voriconazole) have been the most successful backbone in making large number of antifungal compounds available for clinical use. These agents are effective against *Candida* spp. and other fungal pathogens and attractive due to flexibility of administration through different routes (Nett and Andes 2015). Azoles inhibit sterol 14α-demethylase, an essential enzyme in sterol biosynthesis and converts lanosterol to ergosterol, a vital component for maintaining stability and fluidity of fungal cell membranes. While, polyene antimycotics such as amphotericin B and nystatin act on fungal cell membrane through hydrophobic interactions, sequester membrane sterols thereby causing membrane pores and cell death. The semisynthetic lipopeptides, echinocandins (caspofungin, micafungin and anidulafungin) block fungal cell wall synthesis and are active against *Candida* spp. and *Aspergillus* spp. These drugs inhibit 1,3-*β*-d-glucan synthase enzyme coded by *FKS* family genes essential for synthesis of important cell wall components (1,3-*β*-d-glucan) of various fungi and exhibit fungistatic activity. Allylamine drugs retard fungal growth by inhibiting ergosterol biosynthesis and are commonly recommended for controlling superficial dermatophytoses (Newland and Abdel-Rahman 2009). These drugs include terbinafine and naftifine which inhibits squalene epoxidase that converts squalene to lanosterol. Drugs such as pyrimidine analogue (5-fluorocytosine, 5-FC) are active against *Candida* spp. and *Cryptococcus* spp. 5-FC enters the cells through cytosine permeases and deaminated to 5-fluorouracil which affects nucleic acid synthesis (both DNA and RNA) and eventually impairs protein synthesis. Apart from these primary mechanisms, amphotericin B and miconazole are reported to induce oxidative stress and exhibit enhanced antifungal activity. Additionally, new cellular targets such as inhibition of protein synthesis by sordarins, inhibition of microtubule assembly by griseofulvin and inhibition of calcineurin signaling by triphenylethylenes (Scorzoni et al., 2017) have been reported (Fig. 3).

## 5 Antifungal resistance: a barrier in antifungal therapy

**Fig. 4:**
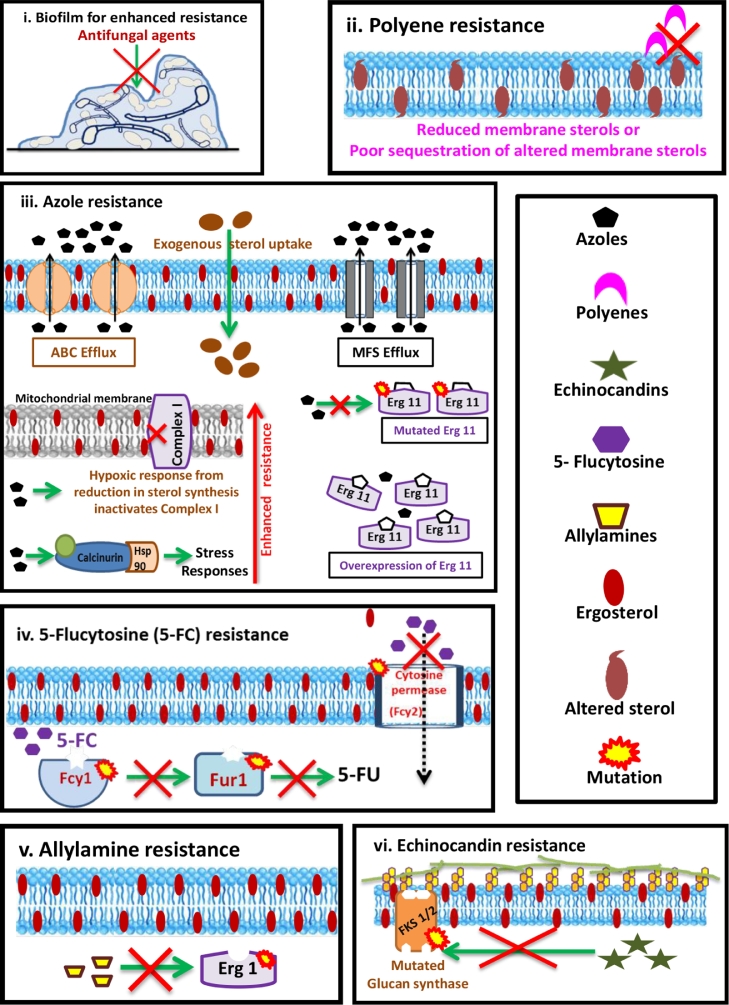
Major resistance mechanisms to common antifungal drugs.

**Fig. 5:**
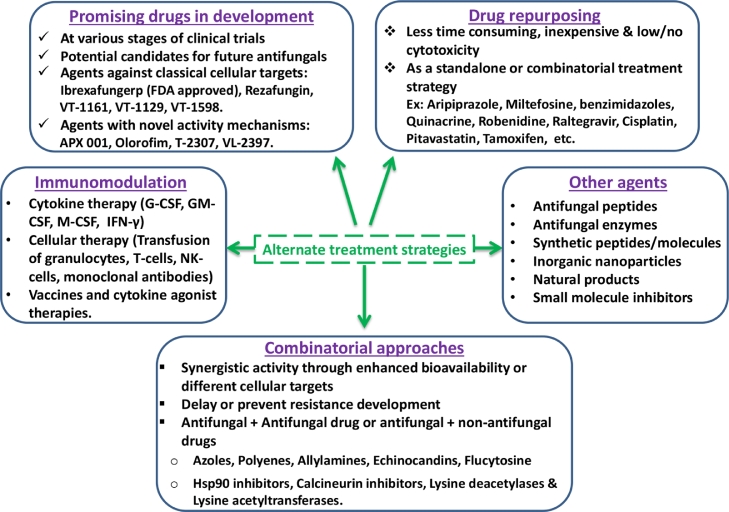
Alternate strategies for effective treatment of fungal infections.

Development of resistance in fungal pathogens to the available drugs is an emerging clinical problem in antifungal therapy (Berman and Krysen 2020). Several host, fungal and environmental factors influence the development of resistance. Adaptive phenotypic plasticity, mutations in target genes followed by selection, chromosomal aneuploidy, sexual reproduction and horizontal gene transfer are the driving forces for emergence of antifungal resistance (Hokken et al., 2019). The major resistance mechanisms conferred by fungi are summarised in Fig. 4. Biofilm formation by *Candida* spp. on medical implants and tissues confer higher resistance to antifungals (Silva et al., 2017; Reddy and Nancharaiah 2020). Fungal biofilms exhibit higher tolerance to drugs due to several factors including their extracellular polymeric substances matrix, slower growth rates, presence of persister cells, exchange of genetic material and synergistic interactions in biofilm cells (Silva et al., 2017; Berman and Krysen 2020). Use of azole drugs is becoming less attractive due to high prevalence of resistance development. The resistance mechanisms include overexpression of ABC (ATP-binding cassette) transporters, MFS (major facilitator superfamily) class of efflux pumps, mutations in *ERG11* or *Cyp51* gene to produce altered target enzyme with low/no affinity to azole drugs and overexpression of ERG11 enzyme (Berman and Krysen 2020). Mitochondrial dysfunction and activation of stress signaling can also contribute to development of resistance to azole drugs (Scorzoni et al., 2017). Although polyenes have been in use for decades, resistance development to polyene antifungals is much lesser as compared to others. Mutations leading to loss of ERG3 function result in low ergosterol content. Decreased ergosterol content and incorporation of altered sterols that have low affinity to amphotericin B are the causes of resistance in *Candida* sp. and *Aspergillus* sp. to polyene drugs (Scorzoni et al., 2017). Fungal resistance to 5-FC is caused by mutations in cytosine permease (Fcy2) and/or Cytosine deaminase (Fcy1), Fur1 enzymes which restrict the entry of 5-FC into the cells and deamination, respectively (Hokken et al., 2019). Mutations in *Erg1* gene encoding squalene epoxidase impair the binding of polyenes causing resistance development. Mutations in echinocandin drug target such as FKS enzyme (glucan synthase) alter conformation thereby decreasing binding affinity and enhances resistance (Scorzoni et al., 2017). Majority of the resistance mechanisms at the genetic level are derived from point mutations or overexpression of genes encoding for target antifungal enzymes/proteins of antifungal agents. Fig. 5

## 6 Alternate strategies for effective antifungal therapy

Limited antifungal arsenal and well documented resistance against available antifungal agents drive the need for development of alternate and effective strategies for combating fungal infections. Fungi being eukaryotic, antifungal drugs can exhibit toxicity to the host. Thus, mammalian cytotoxicity of prospective antifungal agents is an important criterion in drug development. Several strategies have been proposed for developing effective antifungal therapies. The foremost approach is the development of novel agents specific to fungal cells. Potential antifungal agents such as olorofim, VT-1129, VT-1161, VT-1598, CD101, APX-001, T-2307, and VL-2397 are under various stages of clinical trials and potential candidates of future antifungals (Wiederhold 2018). The antifungal agents such as isavuconazole and ibrexafungerp have received U.S. FDA clearance in 2015 and 2021, respectively for treating fungal infections (Van Matre et al., 2019; Schwebke et al., 2021).

Compared to single drug-based monotherapy, synergistic combination therapy enhances drug effectiveness and reduces the emergence of drug resistance. Several *in vitro* and *in vivo* studies reported effectiveness of combinatorial approaches in treating fungal infections (Spitzer et al., 2017; Vitale 2021). The synergistic activity in combinatorial approaches is achieved by the use of drug combinations which have different cellular targets or through the enhancement of bioavailability. The drugs used in combination therapy are by combining two known antifungal drugs or by combining an antifungal drug with a non-antifungal drug. The non-antifungal drugs in combination therapy are purposed for improving the efficacy of antifungal drugs. For example, molecules such as inhibitors of Hsp90 (ex: 17-AAG), calcineurin (ex: cyclosporine A and FK506), lysine deacetylases and lysine acetyltransferases (ex: trichostatin A) are shown to potentiate the activity of antifungal drugs (Spitzer et al., 2017).

Generally, development of newer antifungals involves several years of research and development and associated with extended timelines. This can be overcome by repurposing non-antifungal drugs from the approved drug repositories. This is achieved by computational modeling or docking approaches for screening/mining of molecules with potential antifungal activity followed by experimental validation (Kim et al., 2020). Recent studies based on this approach have identified potential antifungal activity with known drugs such as anti-cancer drug (Tamoxifen), anti-rheumatic drug (auranofin), calcium channel blockers (Nisoldipine, nifedipine, felodipine), anti-inflammatory drug (Asprin, ibuprofen, tacrolimus), and cardiovascular drug (Atorvastatin) (Das et al., 2021). Other repurposed drugs such as aripiprazole, miltefosine, benzimidazoles, quinacrine, robenidine, raltegravir, cisplatin, and pitavastatin were shown to inhibit hyphal induction, biofilm formation and offer therapeutic benefits in infected animal models (Kim et al., 2020).

Modulation of host immunity to fight against invasive fungal infections can be a promising strategy for antifungal therapy. In infected animal models, use of adjuvants like granulocyte colony stimulating factor (G-CSF) and granulocyte-macrophage colony stimulating factor (GM-CSF) improved the efficacy of antifungal drugs in treating *Candida, Aspergillus* and *Cryptococcus* infections (Scriven et al., 2017; Sam et al., 2018). In invasive aspergillosis animal models, IFN-γ enhanced the response to anti-fungal drugs thus improving the survival. In *Candida, Aspergillus, Cryptococcus* infected animal models, administration of fungal cell surface specific monoclonal antibodies enhanced their survival (Scriven et al., 2017). Improved survival in models of invasive fungal infections was reported upon transfusion of granulocytes, T-cells and natural killer (NK) cells (Sam et al., 2018). Live attenuated fungal cells or cell wall components as vaccination strategy has been shown to elicit adaptive immune responses and protection from fungal infections in animal models (Scriven et al., 2017). Additionally, immunomodulation through probiotics, vitamins and microbiome is a promising strategy for future antifungal therapy.

In addition to these strategies, several antifungal peptides, antifungal enzymes, nanoparticles, synthetic chemicals, small molecule inhibitors, natural products and essential oils have shown promising potential as alternate antifungal agents (Padmavathi et al., 2020; Das et al., 2021). However, the use of these agents as standalone or adjuvant antifungal agents should be validated by appropriate assay systems and mammalian cytotoxicity data.

## CRediT authorship contribution statement

**G.Kiran Kumar Reddy:** Conceptualization, Writing – original draft. **Alwar Ramanujam Padmavathi:** Conceptualization, Writing – original draft. **Y.V. Nancharaiah:** Writing – review & editing, Supervision.

## Declaration of Competing Interest

The authors declare that they have no known competing financial interests or personal relationships that could have appeared to influence the work reported in this paper.
